# Understanding Social Determinants of Health and Cognitive Impairment Risk in Diverse Populations Using Machine Learning: A HABS‐HD Study

**DOI:** 10.1002/alz70860_102363

**Published:** 2025-12-23

**Authors:** Ibshar Khandakar, Kai Zhang, Leigh A. Johnson, Edna Patricia Mendoza, Lubnaa B Abdullah, James Hall, Rajesh Nandy, Shanshan Wang, Sid E. O'Bryant, Nicole Phillips, Robert Barber, Zhengyang Zhou

**Affiliations:** ^1^ University of North Texas Health Science Center, Fort Worth, TX, USA

## Abstract

**Background:**

Previous studies have linked certain social determinants of health (SDoH) to Alzheimer's disease and related dementias (ADRD). However, most studies have been focused on individuals of European ancestry, leaving a critical need to investigate the impact of SDoH in other understudied populations. This study examines the impact SDoH and related risk factors on cognitive impairment among three racial/ethnic populations: African American (AA), Mexican American (MA), and non‐Hispanic White (NHW).

**Methods:**

This observational population‐based study assessed 37 SDoH and related factors among a total sample of 3,229 participants from the Health and Aging Brain Study – Health Disparities (HABS‐HD) cohort. Machine learning methods, eXtreme Gradient Boosting and random forests, were used to assess the importance of these risk factors based on their effect on predictive model performance, with the outcome of interest being cognitive impairment diagnosis. The overall population was stratified by each racial/ethnic group (i.e., AA, MA, and NHW) to determine race/ethnicity‐specific associations. The Shapley Additive exPlanations (SHAP) approach was used to determine the marginal contributions for each variable assessed across the models.

**Results:**

Income was among the top risk factors for all three groups. Excluding income, the next top three ranked predictors for the AA population were education, score for the Penn‐State Worry Questionnaire (PSWQ), and national ranking of the area deprivation index. For the MA group, the top risk factors were score for the Geriatric Depression Scale (GDS), score for the PSWQ, and years lived in the United States. For the NHW population, the most important factors were score for the GDS, and self‐reported health status.

**Conclusion:**

The study provides direct evidence of the varying importance of SDoH across a multi‐ethnic community. We highlight communities at the highest risk for cognitive impairment based on SDoH factors. These findings support the implementation of ethno‐racially specific intervention strategies that combine public health initiatives with comorbid disease management to prevent or delay the onset of ADRD in the United States.

